# SAF: Smart Aggregation Framework for Revealing Atoms Importance Rank and Improving Prediction Rates in Drug Discovery

**DOI:** 10.1021/acs.jcim.4c00107

**Published:** 2024-05-02

**Authors:** Ronen Taub, Yonatan Savir

**Affiliations:** Department of Physiology, Biophysics & Systems Biology, Medicine Faculty, Technion IIT, Haifa 3525422, Israel

## Abstract

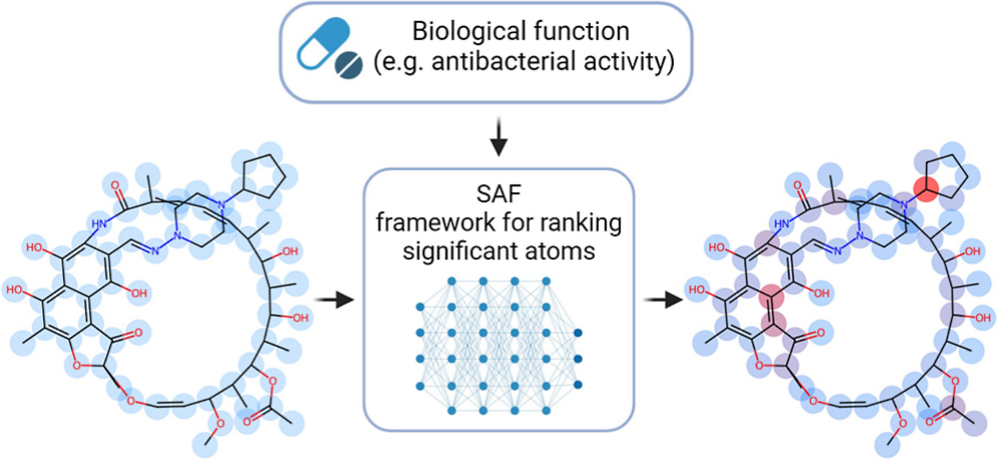

Machine learning, and representation learning in particular, has the potential to facilitate drug discovery by screening a large chemical space in silico. A successful approach for representing molecules is to treat them as graphs and utilize graph neural networks. One of the key limitations of such methods is the necessity to represent compounds with different numbers of atoms, which requires aggregating the atom’s information. Common aggregation operators, such as averaging, result in a loss of information at the atom level. In this work, we propose a novel aggregating approach where each atom is weighted nonlinearly using the Boltzmann distribution with a hyperparameter analogous to temperature. We show that using this weighted aggregation improves the ability of the gold standard message-passing neural network to predict antibiotic activity. Moreover, by changing the temperature hyperparameter, our approach can reveal the atoms that are important for activity prediction in a smooth and consistent way, thus providing a novel regulated attention mechanism for graph neural networks. We further validate our method by showing that it recapitulates the functional group in β-lactam antibiotics. The ability of our approach to rank the atoms’ importance for a desired function can be used within any graph neural network to provide interpretability of the results and predictions at the node level.

## 1 Introduction

One of the promising current trajectories in drug discovery is harnessing the power of machine learning to screen for billions of compounds in silico.^1−5^ The main advantage of this approach is that it requires a reasonable experimental effort to produce a training set on the order of only thousands of compounds. One of the key challenges in applying machine learning for drug discovery is representation of the molecule. The classic approach is to define a feature vector composed of properties based on chemical properties, such as molecule descriptors.^6,7^ Once the molecule is represented as a feature vector, the machine can learn which features are associated with some function. In this approach, the definition of the features representing the molecule requires extensive knowledge in biochemistry. Representation learning, a newer approach in which the machine learns not only what are the features that are relevant to a function but also the relevant features that represent the data, has emerged as a powerful tool.^8^

A successful approach for representing molecules is to consider them as a graph and utilize graph neural networks (GNN) such as graph convolutional network (GCN)^9−11^ and message-passing neural network (MPNN).^12,13^ GNNs are designed to learn how to effectively cast the atomic structure graphs of observed molecules onto new feature spaces, generating representative fingerprints that can be fed into common predictors. They are relatively compact networks and generalize well on drug discovery tasks.^13^ In recent years, the implementations of GNNs for drug discovery have been innovated with modifications like graph attention layer (GAT),^14^ pooling layers,^15^ and GNNs self-supervised regulation.^16^

**Figure 1:**
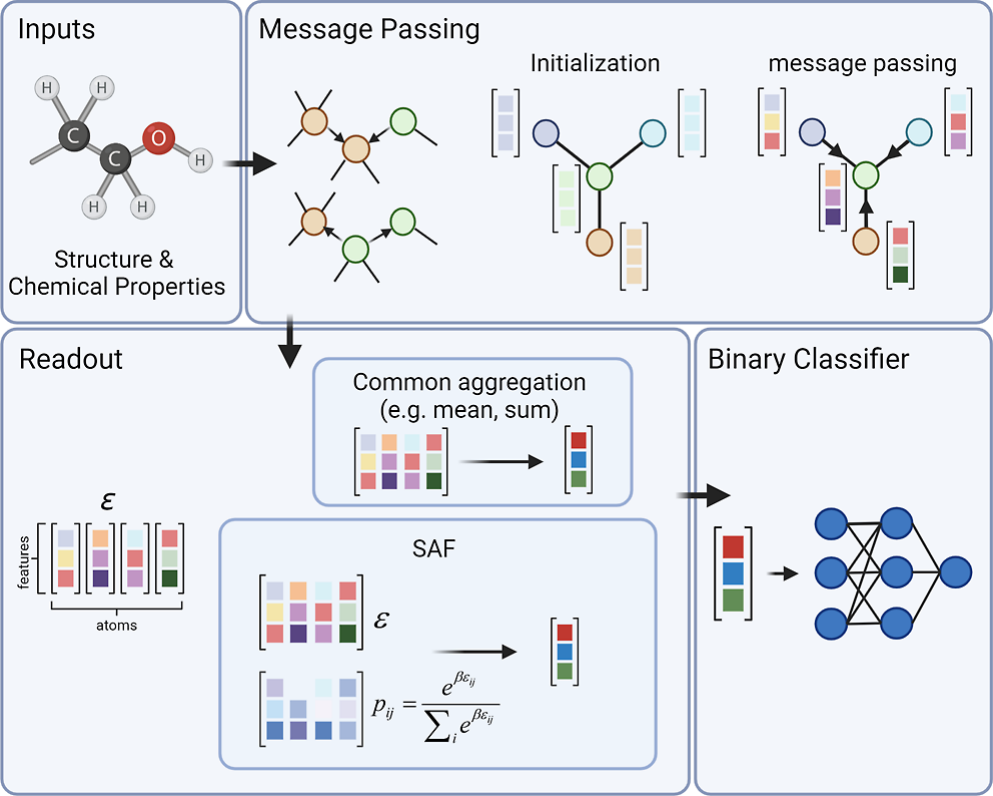
Layout of the MPNN architecture and the SAF operation. The inputs to the network include the molecular structure in the form of a SMILE string and the chemical properties of the atoms and bonds. In the message-passing initialization phase, a feature vector is formed for each node and edge (atom and bond) based on the corresponding chemical properties. In the message-passing phase, the nodes share information over the edges and update constantly based on their neighbor nodes. In the Readout step, the final states of each atom node are extracted, resulting in the encoding matrix ε. Since different molecules have different numbers of atoms, an aggregation operation, such as the commonly used sum, is needed. In the proposed SAF framework, each atom is weighted nonlinearly using the Boltzmann distribution with a hyperparameter, analogous to temperature, β. This allows atom ranking and provides better classification and interpretability.

There are three main steps in the MPNN pipeline: the encoding step, the readout step, and the classification step (Figure 1). In this article, we focus on the readout step. The readout step consists of an aggregative operator that maps the atoms’ encodings , where  and *N*_a_ is the number of atoms, onto a one-dimensional fingerprint vector . The aggregation is mandatory as many classifiers, particularly deep learners, are designed to receive fixed-size vectors as input, while compounds have a varying number of atoms *N*_a_.

The identity of the aggregation operator is a major factor in the model’s performance as it dictates the encoder’s inner states. For example, the most commonly used operator of averaging over all the atoms will transmit only global patterns, traits that all the atoms share. Consequently, the encoder will be biased to create only global patterns, and the binary classifier will be sensitive to only global patterns. On the other hand, single atom aggregators, such as the min(·) and max(·), take into account only local information from one atom. More deterministic operators include var.(·) or standard deviation and the SoftMax operator,^17^ which offers a middle point between global operators and local operators. Besides deterministic operators, there are tunable operators such as GRUAggregation, MLPAggregation, and SetTransformerAggregation.^18^ More sophisticated readout schemes include multiview aggregation, for example, principal neighborhood aggregation,^19^ where the outputs of several deterministic operators are aggregated together for better insights and robustness.

The readout step greatly affects the model’s interpretability. It is the step where the information at the atom level is integrated. This significantly limits the ability to interpret which atoms contribute to the prediction of certain properties and to what extent. Methods to interpret GNN-based pipelines at the node level have been researched for quite some time now using two main approaches: reverse engineering of the converged model or forcing the model to tune down parts of the graphs, implying that the attended parts are important. The first approach includes methods like occlusion sensitivity analysis (OSA),^20^ gradient taping methods, or hidden state maps rescaled by gradients, such as grad-CAM.^21^ OSA methods have been adopted for the use cases of graphs and GNNs. For example, GNNExplainer^22^ and the SME model^23^ implement a masking process in the readout stage to locate important scaffolds and function groups in the case of drug discovery. Despite its simplicity, OSA methods require prior knowledge in the creation of the masks, and there can be differences between the key substructures coming from the literature and formulations coming from the convergence point. Gradient methods are also less appealing as there is a profound question regarding the relation between the convergence point and the true importance factor of each node. The converged model can be partially degenerative due to limitations in the observed data. Also, there could be conceptually wrong microtransactions in the message-passing routine with positive effects.

The second approach includes attention schemes based on GAT.^14,24^ The concept of GAT forces the model to decide to which edge it should attend to. If the prediction performance improves, then this implies that the selected subset of edges is important to the objective. In practice, the attention mechanism in GAT implicitly makes that described interpretability question part of the general objective. Yet, GAT adds a substantial number of new trainable parameters, and therefore, it adds ambiguity to the training routine. In some cases, there can also be conflicts as there could be nodes that are not important for manifesting the property but are important for efficient message-passing communications and vice versa. Most importantly, GAT focuses on ranking edges in the local environment of each node while neglecting the main question of the importance ranking of each node globally (in the molecule).

In this work, we propose a novel readout approach coined the smart aggregation framework (SAF) method that is free of these problems thanks to shifting the attention mechanism into a later stage (not interfering with the GNN encoder). Also, the SAF operation incorporates a hyperparameter that allows the user to observe how the attention (importance) scheme transitions between global and local perspectives in a smooth manner. This offers a more robust and informative view for interpreting and determining the importance of each atom for the learned function. We show that the SAF modification improves the antibacterial prediction performance. To further validate our results, we show that in the case of β-lactam antibiotics, the important atoms converge into the known functional scaffold.

## 2 Methods

### 2.1 SAF: Smart Aggregation Framework

The starting point of the SAF (in short) is the atoms’ encodings available from the GNN encoder (or any other molecule encoder). Let  be the encodings matrix containing the atoms encodings , so that α_*ij*_ is the encoding value of the *i*th atom on the *j*th feature coordinate (where *N*_a_ is the number of atoms and *N*_f_ is the number of features, set to be the hidden state dimension *d*_h_ of the encoder).

The objective of this framework is to sum the elements in α over all of the atoms (rows) for each of the feature coordinates while preserving the local and global patterns. For example, the average operator and the maximum operator are the two extremes of the aggregation operation. The average operator sums the atoms together as equals, while the maximum operator broadcasts a single atom.

The SAF summation operation is given by
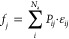
1where ε_*ij*_ is the energy of the likelihood of the *i*th atom to appear in a drug (i.e., positively labeled compound), according to *j*th feature coordinate, and *P*_*ij*_ is a probability-like function that rescales the energy image by comparing atoms. The underlying idea is to amplify local maximum values or minimum values in ε for each feature coordinate, which otherwise would have been deleted by aggregation. We define the discrete probability function *P*_*ij*_ as a Boltzmann distribution
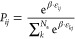
2

For β = 1, the resulting operator in eq 1 is the Softmax operator. *P*_*ij*_ has a statistical mechanics interpretation where β is one over the temperature. In the “hot” case, where β = 0, . In this case, the working point is equivalent to performing the average operation. In the “cold” case, β → ∞, and the resulted operation is max(·) along the atoms coordinate, and if β → −∞, then the resulted operation is min(·) along the atoms coordinate. Hence, the new weighted sum with *P*_*ij*_ as weights, extracts only global patterns (among the atoms) when β → 0, and local patterns only when β → −∞, ∞. For other values of β, we have intermediate working points, which balance in a unique manner between global and local views.

In this work, we set ε_*ij*_ to be α_*ij*_. Thus, when β is sufficiently large, it follows that the model takes into consideration only a subset of the molecule’s atoms. The rest of the atoms are tuned and have a negligible effect on the final fingerprint.

For evaluating SAF, we incorporated the SAF operator in the readout stage of directional MPNN (D-MPNN) architecture and MPNN architecture, with implementations from Chemprop code distribution (GitHub link), and trained for antibacterial prediction.

### 2.2 iSAF: Interpretability with SAF

Interpretability, in terms of finding which atoms are more important for manifesting the targeted property (like antibacterial activity), is crucial for facilitating drug discovery by AI. We define the importance metric, *S*, to be
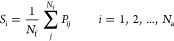
3where *S*_*i*_ is the importance coefficient for the *i*th atom and *P*_*ij*_ is the probabilities from the SAF operation defined in eq 2. The logic is that if the value of *S*_*i*_ is steep, then multiple components of the fingerprint vector *f⃗* will be mainly composed of the *i*th atom encoding (according to eq 1), hence its importance for prediction. It is important to mention that the encodings coming out of the GNN encoder contain only positive entries due to the use of activation functions (specifically, ReLU function), so no mutual cancellation can occur between atoms during aggregation. If β = 0, then  for all *i*, *j*. Meaning, all of the atoms correspond to the same importance coefficient. When β > 0, certain atoms will comply  and others , creating an order among atoms. This is due to the fact that . The case where  for all the atoms (i.e., for all *i*) when β > 0 requires ε_1*j*_ = ε_2*j*_ = ε_3*j*_ = ..., i.e., all ε_*ij*_ are equal for all *i* and single *j* (according to eq 2), which has a negligible probability of occurring.

We extend the metric *S*_*i*_ by looking at the transition over β, i.e., looking at *S*_*i*_(β). A typical discrete matrix  that contains the importance coefficients, i.e., *S*[*i*, *j*] = *S*_*i*_(β = *F*_β_·*j*) (where *F*_β_ is the sampling frequency of β-axis), should have similar properties to the following example matrix
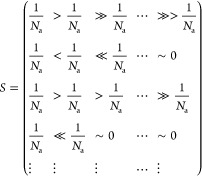


In this example matrix *S*, part of the atoms (rows) have monotonically increasing importance coefficients with respect to β (columns), while other atoms are monotonically decreasing with β. Since  (as shown before), each column vector in *S* has the sum of 1. This means that the monotonic increase in certain atoms’ importance is at the expanse of the other atoms. In the Results section, we show that the given matrix is representative, specifically the property of monotonicity (Results, Section 3.2).

The matrix *S* is the key for ranking the atoms. Analyzing the right portion of the matrix, *S*, yields the most important atoms overall. Analyzing the left portion of *S* allows ranking of the atoms that are less important overall. The distinction between such atoms is lost in the steep values of β. Extracting the trends in the matrix *S* helps to discard artifacts caused by suboptimal training.

To summarize our method, we offer a recipe to interpret molecules at the levels of the atom based on SAF.

1. One should train *N*_β_ model instances for different values of β at each time.
2. calculate the importance coefficients *S*_*i*_(β) out of the rescaling mask *P* from SAF by forward propagation for the targeted molecules.
3. rank the atoms according to *S*_*i*_(β) going backward (from the largest value of β to the smallest value of β). First, one should identify the most important atom, then the second most important atom, and so on.

### 2.3 Preprocessing Routine

The preprocessing routine used to convert the SMILE representation into a graph with initial feature vectors was taken from the Chemprop package (available on GitHub). Each SMILE string was converted into a RDkit molecule object, which gives out its atomic structure and properties. The molecule object was converted into a custom graph object with atoms as nodes and chemical bonds as edges. The graph object contains three main entities: atom nodes features, bond edges features, and connectivity mapping of the graph. The atom nodes features are the initial feature vectors assigned for each node on the graph at the start of the message-passing. Similarly, bond edges’ features contain the feature vectors for the edges. The connectivity mapping is for wiring the messages during the message-passing.

SMILES representations do not include details such as bond angles, bond lengths, or the orientation of subunits in space. RDkit takes into account stereochemical information present in the SMILES notation, such as chiral centers and double bond stereochemistry. It assigns stereochemical properties to the corresponding atoms and bonds in the graph.

The initial feature vector of each atom node consists of the following properties: atomic number, atom mass, atom charge, hybridization, etc. These are all properties of the RDkit::Atom object. Each feature vector created is first discretized and then one-hot encoded to avoid arbitrary numbering systems (such as atomic number). Similarly, the initial feature vector of bonds consists of properties available from the RDkit::Bond object, namely, bond type, ring bond flag, etc. The feature vector for each bond is concatenated to the feature vector of the source atom to the bond, and the result serves as the final initial feature vector of the bond edge.

### 2.4 Model Architecture

Two model architectures were utilized for the evaluation of SAF, MPNN, and D-MPNN. D-MPNN is a modified version of MPNN with the upgrade that each message is subtracted by its corresponding reverse message to avoid saturation of the same information.^12^ The implementation for both was taken from the Chemprop package.

Both MPNN and D-MPNN consist of three parts: the encoder, readout, and prediction. The D-MPNN encoder can be described by the following system of equations
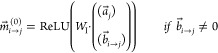
4
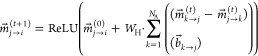
5
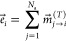
6
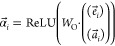
7where  is the initial feature vector of the *j*th atom node (described in the previous section). The indexes of the nodes are arbitrary and derived from the RDkit indexing system.  is the initial feature vector of the bond edge going from the *i*th atom node to the *j*th atom node.

Equation 4 is the initialization phase, where the initial feature vectors are projected onto the hidden space. The result is the messages matrix  with the dimensions *N*_b_ × *d*_h_, where *N*_b_ is the number of bonds in the molecule and *d*_h_ is the hidden space dimension (set to 300 in our case). Equation 5 describes the message-passing phase. The right sum is carried for the bond that contributes to the *j*th atom in the molecule. The addition of  acts as a skipping line to avoid vanishing gradients. Equation 5 is carried *T* times (*T* is set to 2 in our case) to achieve the final messages. In 6 all of the messages are summed for each atom node to get the encodings. Finally, in 7 the encodings are summed together with the original atom feature vectors to get the final encodings (acting as a second skipping line). The size of the final encodings matrix  is *N*_a_ × *d*_h_. From here, the encoding matrix is aggregated according to the readout operation and then summed with the predictor. Here, we replace the aggregation operator with SAF. Instead of averaging over α across the first axis, we apply eq 1 when ε = α. The MPNN encoder offers a simpler scheme and can be described as several GCN encoders chained together.

It is important to note that although the readout matrix is composed of the atoms’ hidden states, it also contains the edge information and the graph connectivity information. This information was passed during the message-passing phase and was embedded inside the atoms’ hidden state. Since we keep the number of message-passing transactions to a small number (2–3 iterations), according to eq 5, it follows that each atom encoding will contain information only on its close surroundings. Consequently, when we rank the atoms with SAF, we rank based on information from substructures centered around each atom.

The model instances we generated were randomly initialized with Xavier initialization which dictates that the generator distribution shall be
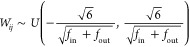
8where *f*_in_ is the number of features fed to the current layer and *f*_out_ is the number of features outputted by the layer. This initialization technique helps to mitigate scaling differences in gradients for different layers caused by differences in the dimensionality of weight matrices from different layers.

### 2.5 Training Routine

Our training routine consists of the binary cross entropy function as a loss function and Adam optimizer with an initial learning rate of 10^–4^ (both from PyTorch.nn implementations). The learning rate scheduler is customized, and its mechanism was originally introduced in the work “attention is all you need”.^25^ The scheduler linearly increases the learning rate at each batch according to a predetermined rate of . After reaching the maximum learning rate, the scheduler decreases the learning rate exponentially until reaching the final learning rate set. From there, it stays constant. The maximum learning rate is set to 10^–3^, and the final learning rate is set to be the initial learning rate. This technique allows the optimizer to “burst” out of the initial environment given by initialization.

To account for variability in the performance benchmarks, we performed 20 cross-validations. We included two types of data splitting; random splitting and scaffold clustering splitting. The scaffold clustering split was carried out using the implemented routine in the Chemprop package, which utilizes Murcko Scaffold from Rdkit::Chem::Scaffolds to cluster the compounds and then allocates the clusters to a training set, validation set, or test set. The ratio between sets is 8:1:1 (when the training set is 80%). We trained on the training set for 30 epochs with two warmup epochs. Upon the end of optimization, we took the model instance that corresponds to the epoch with the maximum AUROC (area under the ROC curve) value for the validation set. In the test, we tested the chosen model instance and calculated the AUROC value for the test set.

In the evaluation, all of the models were trained in the same described fashion. The changes between the runs were made in the readout operator for comparison. In the case of SAF(β), we optimized β as a model parameter. Since β is encapsulated in an exponent function, the scale of the gradients for β is not comparable to the rest of the model. Therefore, we implemented a separate learning rate scheduler for the gradients for β. The scheduler is customized to output a zero learning rate for a single epoch, raise linearly to a predefined maximum learning rate (set to 1) during the second epoch, and then decrease exponentially to the final learning rate (set to 10^–4^). During the first epoch, the model is trained as if β = 0 to generate sensible encodings at the end of the epoch. At the second epoch, β is optimized with a greater learning rate than that for the rest of the model, based on the encodings developed in the first epoch. Afterward, the entire system “cools off” with significantly lower learning rates.

As part of benchmarking the interpretability and atom ranking of SAF, we trained a reference model for scaffold ranking termed the SME model.^23^ The SME model implements an OSA method in the readout phase by masking the atoms’ encodings that belong to a known scaffold. We took the source code provided with the published work and trained an SME model instance with the original training routine and recommended hyper-parameters. The model consists of an ensemble of 10 GCNs. After training the SME model on the *E. coli* data set, we used the explainability routines provided to extract the attribution for predetermined scaffolds. To produce atom rankings, we repeated the process for scaffolds with a single atom for each atom in the molecule and ranked the resulting attribution scores.

### 2.6 Data Sets

The *E. coli* data set consists of 2335 compounds that were screened for growth inhibition against *E. coli* to create a data set for drug screening AI training.^13^ This data set contains 1760 FDA-approved molecules of diverse structure and function and 575 natural products isolated from plant, animal, and microbial sources. As described in the paper,^13^ the final screening concentration was 50 μM. They used the 80% growth inhibition as a hit cutoff; this primary screen resulted in the identification of 120 molecules with growth-inhibitory activity against *E. coli*. The final data set contains SMILE strings, corresponding growth inhibition marks, and binary classification from the threshold.

The Bioactive data set contains the compounds union between the Drug Repurposing Compound Library (HY-L035P, MedChemExpress) and the Bioactive Compound Library (HY-L001P, MedChemExpress) that have SMILE information in the PubChem database. This data set contains 5132 approved and clinical drugs passing phase I and small molecules with validated biological and pharmacological activities. Bioactivity information, including target and clinical data, is available for these compounds from the library manufacturer. 251 compounds are categorized as having antibiotic activity.

The two data sets’ compounds do not intersect.

## 3 Results

### 3.1 Using SAF Improves Antibiotic Activity Prediction

We evaluated the SAF performance for antibacterial activity prediction. We used two mutually exclusive data sets. The *E. coli* data set contains the effect of 2335 FDA-approved compounds on the bacteria *E. coli*, and the Bioactive data set contains 5132 bioactive compounds, labeled for antibacterial activity (Methods, Section 2.6). We compared the AUROC (area under the ROC curve) metric for different aggregation operators in the readout step and GNN architectures in both data set cases. We examined in each case both D-MPNN and MPNN (Methods, Section 2.4). In the case of SAF, we optimized β as a model parameter together with the rest of the model (Methods, Section 2.5). We focus on the AUROC metric since the final model in each run corresponds to the maximum AUROC value for the validation set (across epochs). The benchmarks are given in Table 1.

**Table 1:** Antibacterial Activity Prediction for Different Aggregation Operators in the Readout Step^a^

	*E. coli* data set		bioactive data set
aggregation method	MPNN	D-MPNN	D-MPNN
SAF(β)	0.8879 ± 0.0469^1^	0.9001 ± 0.0373^1^	0.8151 ± 0.0285^1^
min.	0.8816 ± 0.0578^2^	0.8962 ± 0.0404^2^	0.7738 ± 0.0414
max.	0.8699 ± 0.0663	0.8894 ± 0.0568^3^	0.8056 ± 0.02^2^
mean	0.84 ± 0.0729	0.8435 ± 0.0678	0.7701 ± 0.0146
sum.	0.8322 ± 0.0914	0.83 ± 0.0825	0.7983 ± 0.0277^3^
mul.	0.8678 ± 0.0607	0.7429 ± 0.1072	0.7 ± 0.0867
std.	0.8709 ± 0.0628^3^	0.8661 ± 0.0767	0.804 ± 0.0204^3^
SoftMax	0.8425 ± 0.0822	0.837 ± 0.0766	0.7773 ± 0.037
GRU	0.8231 ± 0.0769	0.8179 ± 0.0766	0.7819 ± 0.0254
attention	0.8446 ± 0.0707	0.819 ± 0.09	0.7475 ± 0.0341
SME	0.8152 ± 0.0912	0.8335 ± 0.0801	0.7889 ± 0.0426

a SAF(β) denotes training with β as the model parameter. GRU denotes GRUAggregation from Pytorch-Geometric. Attention refers to SetTransformerAggregation from Pytorch-Geometric. SME denotes the aggregation operator implemented in the SME model.^23^ The subscript numbers denote the ranking of the top-3 operators.

To account for variability in the training data, we performed a large number of comparisons. We carried out 20 unique cross-validations for each combination with a training-validation-test splitting ratio of 8:1:1. We compared ten aggregation operators in the readout stage, along with SAF (Table 1). We excluded aggregation methods that utilize multiview aggregation as they can include SAF as a view or be emulated by SAF with a vector of different β values. The results in Table 1 are for the random splitting. We also included the results for scaffold splitting (Methods, Section 2.5) in Supporting Information (Section 2.1 and Table S1). The results in Table 1 indicate that SAF(β) shows top performance, which is robust to different architectures and different data splitting.

**Figure 2:**
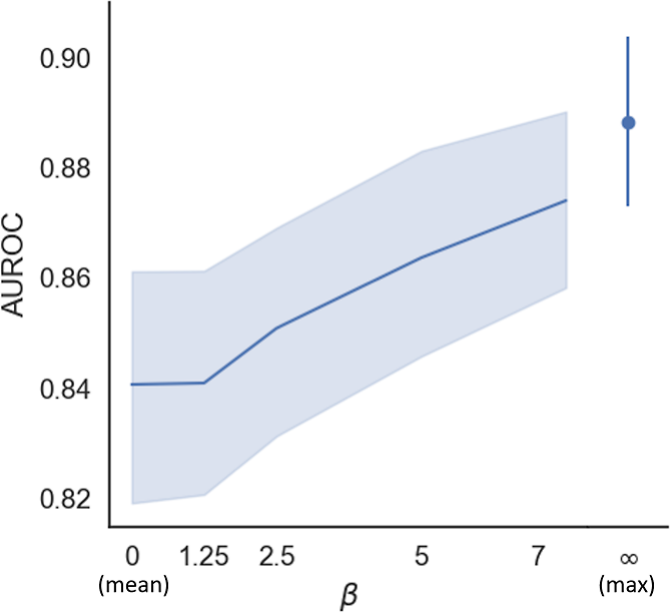
Average AUROC (area under the ROC curve) for predicting antibiotic activity as a function of β over 20 different data set splits of the *E. coli* data set. The error bars are the 95% confidence interval.

To better understand the connection between β and prediction performances, we analyzed the effect of β on the ability of D-MPNN to predict antibiotic activity over a grid. We ran 20 unique cross-validations on the *E. coli* data set for each point on the grid. Figure 2 shows the average behavior with the AUROC metric distribution for each point on the grid. The point labeled as “MAX” on the grid marks the case where β → ∞, which is not feasible to calculate with SAF but can be calculated with max(·), which is equivalent.

The results indicate that as β increases, the AUROC metric increases monotonically. This observation is significant because as β increases fewer atoms contribute to the final prediction. To make sure that this result also holds for unseen data, we used the 20 converged models for each point on the grid (which are trained on the *E. coli* data set) and extracted the prediction for the entire Bioactive data set. Since both data sets are mutually exclusive, the compounds in this data set act as unseen data. The results are presented in the Supporting Information (Section 2.1 and Figure S1) and indicate that while, as expected, the absolute AUROC is lower, the increase AUROC with β is not data set-specific.

### 3.2 Monotonicity of Important Atoms

**Figure 3:**
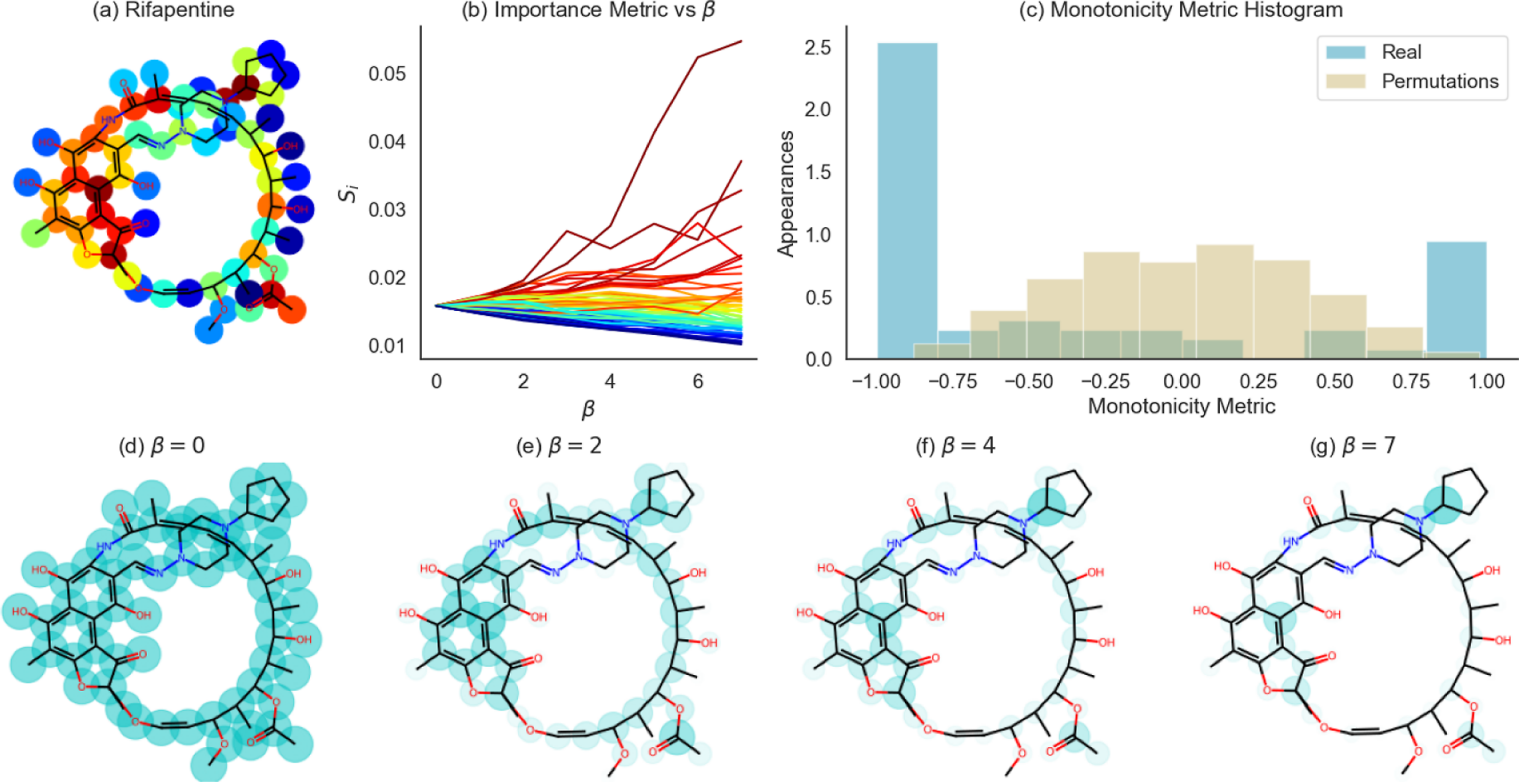
(a) Atomic structure of the antibiotic rifapentine. (b) Importance coefficients, *S*_*i*_, as a function of β for the different rifapentine atoms. The colors of the atoms in (a) and the graphs in (b) are matching for each atom, and the colors represent the importance of each atom, *S*_*i*_(7). (c) Histogram of Spearman’s rank correlation coefficient between the *S*_*i*_ in (b) and linear dependence in β (cyan bars), and histogram of Spearman’s rank correlation coefficient between random permutations of *S*_*i*_ and linear dependence in β (yellow). The observed Spearman’s correlation distribution is significantly different from random dependence on β (*p*-val <0.05, Kolmogorov–Smirnov test). (d–g) Illustration of the smooth and monotonic change in the atom importance. Atomic structures are colored by the *S*_*i*_ coefficients (intensity and radius) for different values of β.

The contribution of each atom is captured by *S*_*i*_(β) (eq 3). In the case of β = 0, all of the atoms have the same weight. As β increases, some atoms have higher weights, and for β → ∞, only one atom contributes. The emerging question is whether the important atoms for different values of β are random or whether the importance order of atoms is kept while increasing β, thus revealing the importance of atoms gradually. In this section, we show that, indeed, increasing the hyperparameter β reveals in a smooth fashion the atom’s importance. Figure 3b illustrates *S*_*i*_(β) for the antibiotic drug rifapentine, an example compound. As β increases, the atoms’ importance quotas emerge in a monotonic and smooth fashion.

To quantify the monotonicity of the importance metric, we used the Spearman’s rank correlation coefficient between *S*_*i*_(β) and a linear function of β. To show that the results are indeed monotonic, we compared the actual *S*_*i*_(β) coefficients with coefficients that resulted from random permutations between different β values. The results indicate a significant monotonicity of *S*_*i*_(β) graphs (Kolmogorov–Smirnov test, *p*-val <0.05) (Figure 3c). Visually, we can see how the Spearman’s rank correlation coefficients in the case of *S*_*i*_(β) are located in the two extreme bins of the histogram in a significant manner. The meaning of the results is that part of the atoms have monotonically increasing importance graphs, while others regress, which is also visible in Figure 3b, conveying what was stated in Subsection 2.2.

In Figure 3d–g, the atomic structure of the molecule is colored according to the importance pattern given by  for different values of β. In the case of β = 0, all of the atoms have the same importance coefficient and are colored with the same intensity and radius (Figure 3b,d). When β increases, the importance pattern converges into a subset of atoms (Figure 3d–g).

**Figure 4:**
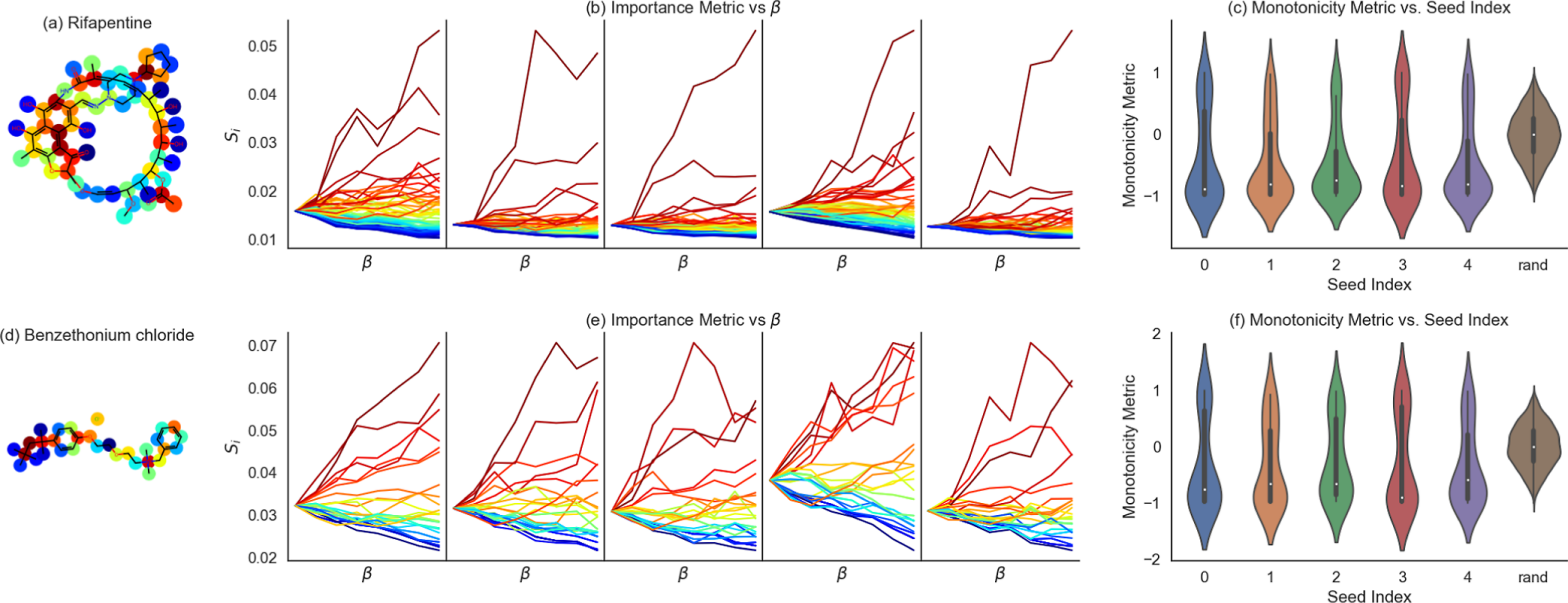
Effect of β on the atom importance for five different data splits for rifapentine (a) and benzethonium chloride (d). (b) Importance coefficients, *S*_*i*_, as a function of β. The colors on the atoms’ structure match the *S*_*i*_ plots and represent the importance parameter, *S*_*i*_(7). (c) Violin plot of the distribution of Spearman’s rank correlation coefficients between the line plots in (b) and a linear dependence in β for 5 different train-validation-test splits (marked with the seed index). The sixth distribution labeled “rand”, is the distribution of Spearman’s rank correlation coefficients between random permutations of the line plots in (b) and a linear dependence in β. (e–f) is the same as that for (c–d) for benzethonium chloride.

Figure 3 shows the rifapentine’s results for a single train-validation-test split. Next, we want to show that similar results (with respect to monotonicity) are received for different molecules and data set splits. To show the monotonicity is not a one-time result of a particular data set split, we randomly selected 6 molecules out of the data set and randomized 5 train-validation-test splits where all 6 selected molecules appear in the randomized test sets. So, in total, we have 5 instances for comparison (Figure 4). Besides rifapentine, Figure 4 also shows the results for benzethonium chloride. The results of all six molecules appear in Figure S3 of the Supporting Information

For all the different data splits, there is a significant monotonicity of the atoms’ importance (Figure 4c,f).

### 3.3 Reproducibility of Atoms Rankings with iSAF

**Figure 5:**
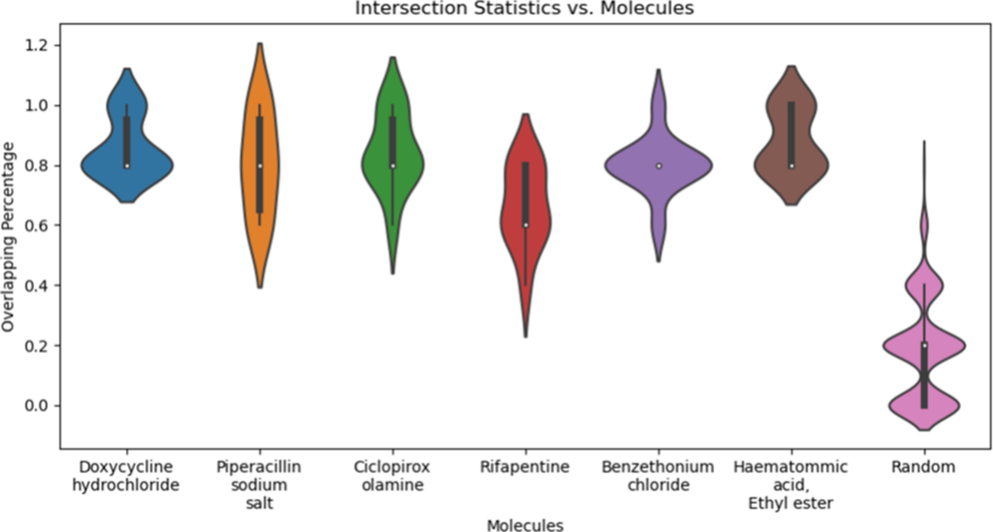
Violin plot of the distributions of the fraction of overlapping atoms among the top-5 most important atoms for β = 7 from different seeds. The last distribution labeled as “Random” is the distribution of overlapping atoms when the ranking of atoms at each seed is randomized. The overlap distributions for all the molecules are significantly higher than expected by random (*p*-val <0.05, Kolmogorov–Smirnov test).

When exploring different data set splits, a very relevant metric is the size of the overlap between the atoms’ orders. That is, the ranking order of the atoms does not fluctuate between different β values. To quantify the order’s smoothness, we calculated the number of overlapping atoms among the top 5 most important atoms according to *S*_*i*_(β = 7) in different seeds. In Figure 5, we show the distribution of the number of overlapping atoms when comparing pairs of different train-validation-test splits (for the 6 molecules in the test set). In Figure 4b, section e, we can see the overlap visually.

There is a significant overlap in the top-ranked atoms with an average over 80% (4 out of 5 atoms appear in the intersection). The same cannot be said for the random noise. We generated the random samples by permuting the *S*_*i*_(β) sequences (similar to before) and taking the top-5 atoms from the case of β = 7. The resulting *p*-value is significantly smaller than the 0.05 mark (Kolmogorov–Smirnov test). Therefore, not only are the importance coefficients *S*_*i*_ very consistent over β (due to monotonicity), but also the identity of the important atoms based on them is consistent over different seeds and for different molecules.

### 3.4 iSAF Recapitulates β-Lactam Antibiotics Functional Group

**Figure 6:**
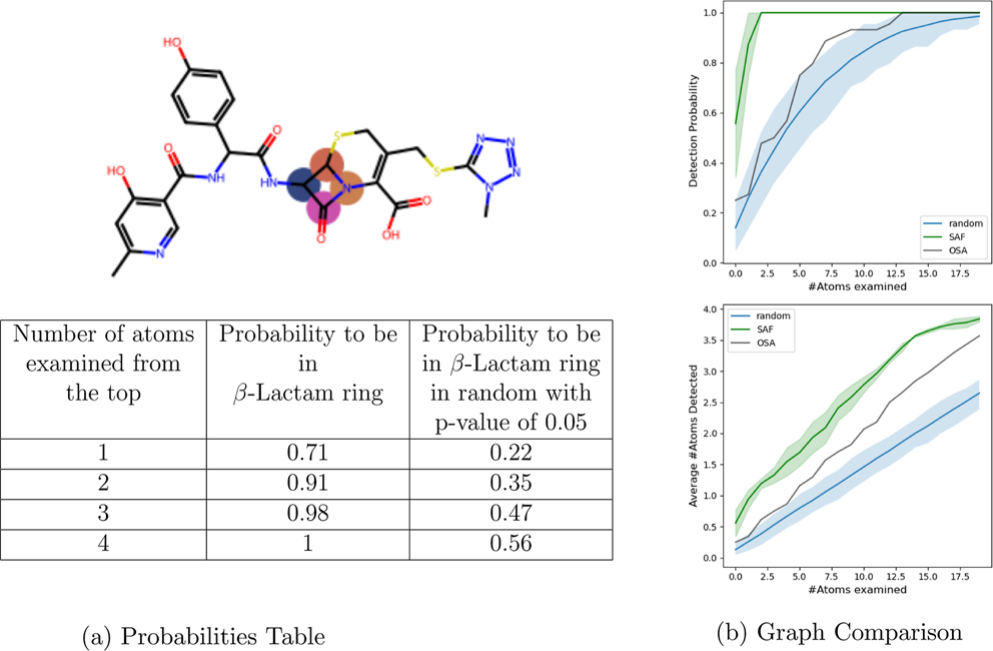
(a) Fraction of β-lactam antibiotics that at least one of their top-ranked atoms, for β = 7, appears in the β-lactam ring. The left column is the number of top atoms considered. For example, for all 44 β-lactam antibiotics in 68 cases, at least one of the 4 top-ranked atoms was within the β-lactam ring. The right column contains the fractions of at least one out of a random set of atoms within the β-lactam ring, where the random set is sampled from a random event with a probability of 0.05 (for each molecule). (b) Two comparisons of the atom ranking performances between SAF (green) and an OSA method, the SME model (black), and random ranking (blue). The blue shaded area is the 95% confidence area. In the first comparison, the probability of at least one top atom being in the β-lactam ring is compared as a function of the total number of atoms examined from the top-ranked ones. The second comparison shows the number of atoms from the β-lactam ring detected as a function of the total number of atoms examined from the top-ranked ones.

To validate the functional relevance of the emerged importance coefficients, *S*_*i*_(β), we focused on β-lactam antibiotics, which include Penicillin derivatives, as an example. These antibiotics have a substructure that accounts for the antibacterial activity known as the β-lactam ring (Figure 6). We aimed to validate whether the top-ranked atoms, coming out of the *S*_*i*_(β), do reside within the β-lactam ring. Hence, we collected all the β-lactam antibiotic compounds from the *E. coli* data set, amounting to 44 unique molecules that can be examined. For each compound, we calculated the *S*_*i*_(β = 7) out of SAF operation in forward propagation with the models from different cross-validations. Then, we counted the number of cases in which the topmost important atoms reside within the β-lactam ring out of 68 cases. We did the counting for the first most important atom only, the two most important atoms, the three most important atoms, and the four most important atoms according to *S*_*i*_(β = 7). The results are presented in the table in Figure 6a.

The results indicate that the representation of the β-lactam ring within the important atoms is significantly higher than what is expected randomly. All 68 cases have at least one significant atom from the top 4 atoms in the β-lactam ring. Looking at only the single most important atom in each case, 71% of the cases had it inside the β-lactam ring. Interestingly, the atoms from the top that do appear in the ring are (with high frequency) the same atom in the configuration of the β-lactam ring (colored with orange). The bias for the same atom may shed some light on the GNN encoder’s view of scaffolds. It is insightful to examine two metrics for the quality of atom ranking. The first is the probability of finding at least one atom from the β-lactam ring within the top-ranked atoms (Figure 6a), and the second is the average number of β-lactam ring atoms within the top-ranked atoms (Figure 6b). Our results show that in both metrics, SAF performs significantly better than random ranking. To benchmark our results, we compared iSAF with an OSA method for interpretability, the SME model,^23^ which provides state-of-the-art performance for ranking functional masks. We trained SME on the *E. coli* data set and used the OSA approach to rank the atoms (that is, instead of masking a functional group, a single atom is masked) (Methods, Section 2.5). In both metrics (Figure 6), SAF performs significantly better than SME.

## 4 Discussion

One of the main challenges of applying message-passing neural networks for drug discovery is the need to cope with compounds with a different number of atoms. Thus, aggregating the atoms’ information is a common bottleneck in many applications. The common aggregation approach is to sum over all atoms’ features, and therefore, atoms that are not affected by each other during the message-passing step (which is local) are blended. In this work, we propose a novel aggregating approach where each atom is weighted in a nonlinear fashion using the Boltzmann distribution where the “energies” are the atoms’ features from the message-passing stage and the “temperature” 1/β is a hyperparameter. When β = 0, the system is “hot”, and the aggregation is a simple sum. When β → ∞, the system is “cold”, and the aggregation is the max(·) operator. Thus, controlling β provides a handle over the trade-off between local and global information.

Our results show that as β increases, there is an improvement in the antibiotic classification AUROC. That is, as the network is forced to account for a subset of the overall atoms, the performance improves. Thus, the emerging questions are what are the groups of atoms that are contributing, and whether their identity is robust across different cross-validations and different values of β. As β increases, fewer atoms contribute significantly to the prediction, yet the ranking of the atoms is preserved along different values of β (Figures 3 and 4) and different cross-validation (Figure 5). That is, the increase of β serves as akin to phase separation that amplifies the contribution of the more important atoms in a smooth fashion. Therefore, our approach can be used as a regulated attention mechanism. Unlike other GATs that operate at the message-passing phase by adding more weights that attenuate the message-passing transactions, our approach does not interfere with this phase. This is a crucial point as it allows for atoms that are not necessarily in the same neighborhood on the graph, to emerge as significant atoms.

Another important property of our approach is the ability to reveal the significance at the atom level. To validate our approach, we show that our model highlights the β-lactam ring, the functional group of β-lactam antibiotics, in a statistically significant way. The fact that our schema allows a smooth highlighting of atoms as β increases provides not only interpretability but also a better understanding of the data set composition that could be important for achieving better data splits.

In this work, each atom is weighted nonlinearly using the Boltzmann distribution with a hyperparameter analogous to temperature. This approach provides an intuitive tunable parameter that enables ranking of the atoms. One possible way to enrich the model is to include the analogous of a tunable field in the probability calculation step such that a higher order of interactions between the atoms is accounted for. While other models focus on scaffold rankings, our work focuses on atom rankings. The main advantage of this approach is that it is not limited or biased by a functional group bias. Yet, the ability to incorporate scaffolds that are intuitive building blocks provides a means to interpret chemical function. Future work can introduce functional groups as a prior to SAF and, by that, achieve not only the importance of a particular group but also the contribution of each atom within the functional group to the relevant chemical function. The aggregation operator is considered to be the lesser part of the GNN architecture and is often being neglected. Our work highlights its importance and can be harnessed to other applications of message-passing neural networks.

## Data Availability

All the data is available in machine reading format in the Supporting Information. The data and code are also available at GitFront and in GitHub.

## Supporting Information Available

Data tables description, additional results for scaffold splitting and for the bioactive data set, tSNE plot of Morgan fingerprints from the *E. coli* data set and Bioactive data set, and atom rankings of additional test molecules (PDF)
List of compounds (ZIP)
Compounds’ antibiotics’ prediction (ZIP)
Importance coefficients (ZIP)
Bioactive data set (ZIP)
Top five atoms (ZIP)
Atom ranking β-lactam antibiotics (ZIP)
