# Supplementary material for: SAF: Smart Aggregation Framework for Revealing Atoms Importance Rank and Improving Prediction Rates in Drug Discovery
Source: J Chem Inf Model. 2024 May 2;64(10):4021–30. doi: 10.1021/acs.jcim.4c00107 (PMC11134513; doi:10.1021/acs.jcim.4c00107)
Supplement: Supplementary file 1 — ci4c00107_si_001.pdf [file ci4c00107_si_001.pdf]

Supporting Information

Ronen Taub and Yonatan Savir*

Department of Physiology, Biophysics & Systems Biology, Medicine Faculty, Technion IIT, Haifa, Israel

E-mail: yoni.savir@technion.ac.il

Data Tables

1. `final_dataset_ecoli.csv` - the E.coli dataset used for training.

2. `final_dataset_bioactive.csv` - the Bioactive dataset used for training.

3. `final_figure_2_data.csv` - the data for figure 2 in the paper, contains AUROC values for each training instance.

4. `final_figure_3_4_data.npy` - the data for figure 3 and figure 4 in the paper, containing the S_i importance coefficient for each value of β (shape: 6 molecules X 5 seeds X 8 values of β).

5. `final_figure_5_data.npy` - the data for figure 5 in the paper, containing the distribution of the overlapping atoms between different seeds (shape: 6 molecules X 5 seeds X 5 top atoms).

6. `final_figure_6_data.csv` - the data for figure 6 in the paper, contains the atoms ranking of β-Lactam antibiotics.

Supplementary Figures and Tables

Table S1: Antibacterial activity prediction on the E.coli dataset with Scaffold splitting.

h

Aggregation	E. coli Dataset - Scaffold Split	
Method	MPNN	D-MPNN
SAF(β)	0.8453 ± 0.0824[1]	0.8439 ± 0.0792[3]
Min.	0.8262 ± 0.0705[3]	0.857 ± 0.0601[2]
Max.	0.8406 ± 0.0765[2]	0.8581 ± 0.0729[1]
Mean	0.7982 ± 0.0779	0.7991 ± 0.0853
Sum.	0.78 ± 0.0946	0.775 ± 0.0991
Mul.	0.8125 ± 0.127	0.7234 ± 0.1024
Std.	0.8253 ± 0.0777	0.8172 ± 0.0919
SoftMax	0.8001 ± 0.0812	0.7892 ± 0.0917
GRU	0.7702 ± 0.0834	0.7909 ± 0.1005
Attention	0.812 ± 0.0576	0.7979 ± 0.0796
SME	0.7535 ± 0.1181	0.7762 ± 0.0886

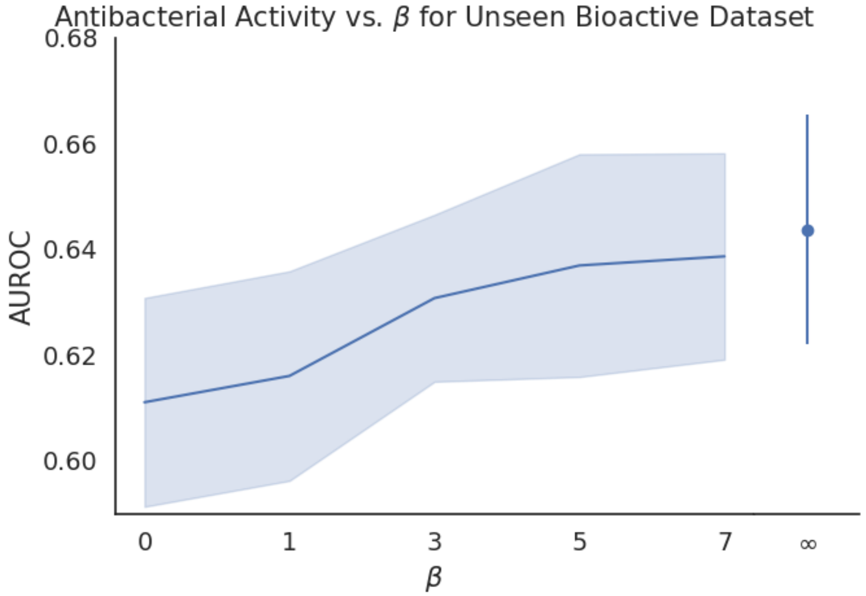

Figure S1: Antibacterial activity prediction statistics for 20 cross-validations when training on E.coli data and testing on Bioactive dataset, an external unseen dataset.

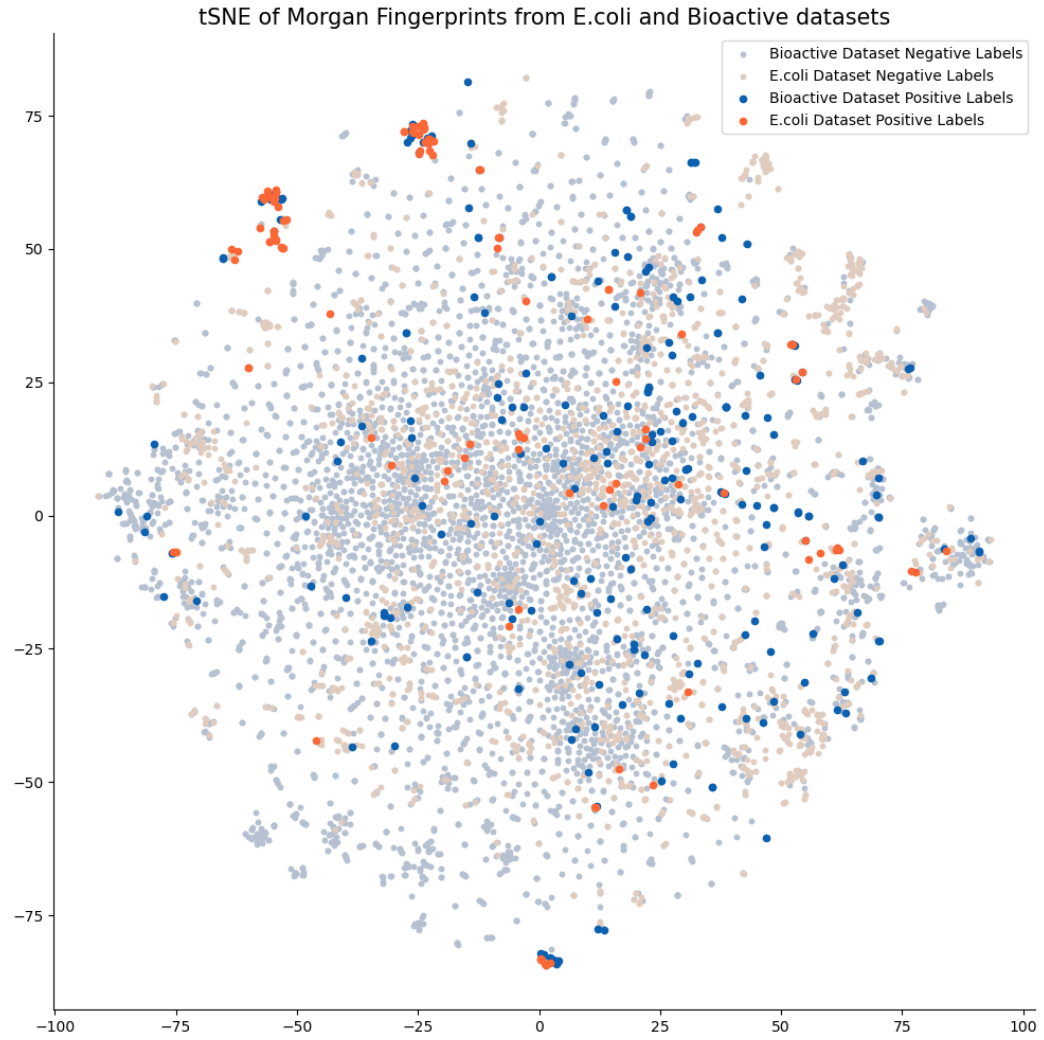

Figure S2: Scatterplots of $2D$ tSNE projection of the Morgan fingerprints encodings for compounds from both datasets. The Morgan fingerprint is a binary encoding technique for encoding compounds' molecular structure. The distribution indicates that while there are several clustered positively labeled compounds, the majority of positively labeled compounds are scattered along the chemical space. This reflects the high degree of difficulty in separating between compounds and predicting anti-bacterial activity based on molecular structure. Zooming in on the clustered areas reveals that there are negatively labeled compounds alongside positive compounds.

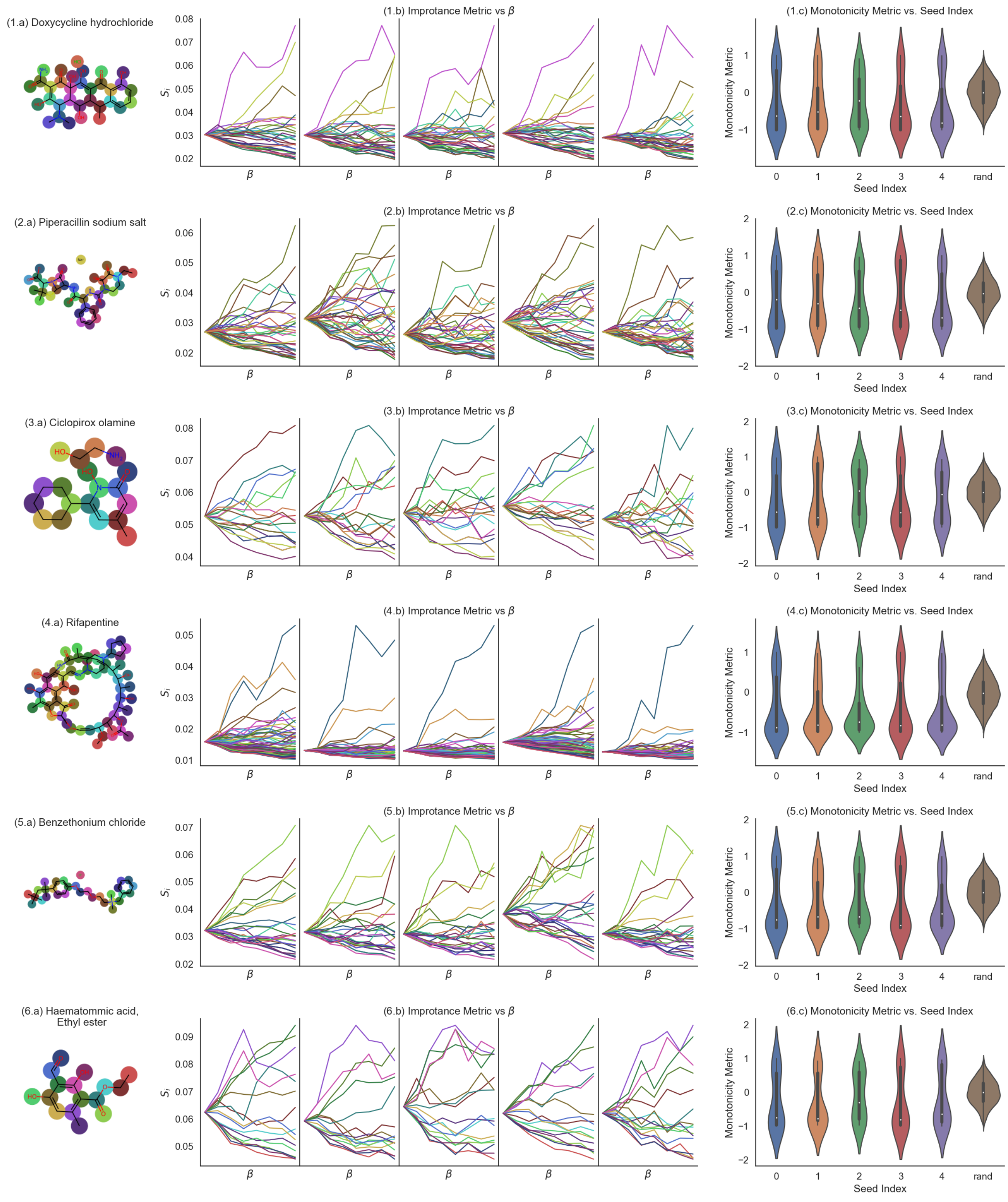

Figure S3: The effect of β on the atom importance for five different data splits and for six unique compounds. (1.a)-(6.a) The colors on the atoms' structure are matching to the S_i plots of panels (b). (1.b)-(6.b) The importance coefficients, S_i, as a function of β. (1.c)-(6.c) Violin plots of the distribution of Spearman's rank correlation coefficients between S_i and a linear dependence in β, for 5 different train-validation-test splits (marked with the seed index). The sixth distribution labeled 'rand' is the distribution of Spearman's rank correlation coefficients between random permutations of the line plots in panels (b) and a linear dependence in β.
